# Roles of microRNA‐22 in Suppressing Proliferation and Promoting Sensitivity of Osteosarcoma Cells *via* Metadherin‐mediated Autophagy

**DOI:** 10.1111/os.12442

**Published:** 2019-04-01

**Authors:** Peng Wang, Zhen‐qun Zhao, Shi‐bing Guo, Tie‐yi Yang, Zhi‐qiang Chang, Dai‐he Li, Wei Zhao, Yu‐xin Wang, Chao Sun, Yong Wang, Wei Feng

**Affiliations:** ^1^ Orthopedics Department Second Affiliated Hospital of Inner Mongolia Medical University Hohhot China; ^2^ Orthopedics Department, Inner Mongolia Institute of Orthopaedics Hohhot China

**Keywords:** Autophagy, Chemoresistance, MicroRNA‐22, Metadherin, Osteosarcoma

## Abstract

**Objective:**

To analyze the effect of microRNA‐22 on autophagy and proliferation and to investigate the underlying molecular mechanism of osteosarcoma cell chemotherapy sensitivity.

**Methods:**

MG‐63 cells were divided into four groups, including a control group, a negative control (NC) group, a cisplatin group, and a cisplatin + miR‐22 group. Proliferation of MG‐63 cells that had been treated with cisplatin and transfected with miR‐22 mimics was determined using MTT assay and colony formation assay. We assessed the degree of autophagy using flow cytometry through cellular staining of the autophagy lysosomal marker monodansylcadaverine (MDC). The effect of microRNA‐22 on autophagy was observed along with the expression levels of Beclin1, LC3, metadherin (MTDH) and ATG5 by western blot and quantitative reverse transcription polymerase chain reaction (qRT‐PCR). Luciferase reporter assay revealed the targeted binding site between miR‐22 and the 3′‐untranslated region (3′‐UTR) of MTDH mRNA. Western blot and qRT‐PCR were used to explore the level of MTDH in the control group, the NC group, the cisplatin group, and the miR‐22 group for 6, 12, and 24 h.

**Results:**

In the *in vitro* study, the MTT results indicated that the MG‐63 cells with overexpression of miR‐22 exhibited a significant decline in the proliferation capacity compared with the control group (0.513 ± 0.001, *P* < 0.0005). Similar to the MTT results, MG‐63 cells that were transfected with miR‐22 mimic (101.0 ± 10.58) formed fewer colonies compared with the cisplatin group (129.7 ± 4.163). MDC staining revealed that miR‐22‐overexpressing osteosarcoma (OS) cells treated with cisplatin showed a significant decrease in the expression of autophagy (7.747 ± 0.117, *P* < 0.0001). Our data revealed that miR‐22 could regulate not only autophagy but also proliferation in the chemosensitivity of osteosarcoma cells. We found that miR‐22 sensitized osteosarcoma cells to cisplatin treatment by regulating autophagy‐related genes. In addition, Luciferase Reporter Assay revealed that miR‐22 negatively regulated autophagy through direct targeting of MTDH. We performed western blot analysis to detect the MTDH expression level. The results revealed that the overexpression of miR‐22 obviously decreased the expression of MTDH (1.081 ± 0.023, *P* < 0.001).

**Conclusion:**

Inhibition of miR‐22 ameliorated the anticancer drug‐induced cell proliferation decrease in osteosarcoma cells. MTDH was identified as the miR‐22 target in OS cells and MTDH‐triggered autophagy played a key function in the miR‐22‐associated chemotherapy sensitivity.

## Introduction

Osteosarcoma (OS) is a robust malignant cancer of the bone affecting children and adolescents^1^. Over the past several decades, application of adjuvant chemotherapy, such as cisplatin, doxorubicin, and methotrexate, has led to a dramatic increase in the survival rate of osteosarcoma^2^. Nevertheless, like most cancers, some patients will develop relapse and chemoresistance. The frequent acquisition of drug resistance is often associated with chemotherapy and is a significant obstacle to achieving a favorable outcome^3^. Studies have demonstrated that numerous mechanisms mediate the development of chemoresistance, including DNA repair^4^, altered drug accumulation^5^, autophagy‐related chemoresistance^6^, and micro‐RNA (miRNA) dysregulation^7^. Thus, to overcome this significant obstacle, research into the mechanism of chemoresistance of OS is important.

Autophagy is a degradation pathway of lysosome‐dependent organelles or cytoplasmic components, and the basic process of cells maintaining their internal environment^8^. It has also been established as a cytoprotective process that is induced by environmental stresses, including hypoxia, metabolic stress, as well as chemotherapy. Extensive research suggests that autophagy is critically involved in the development of cancer and cancer chemoresistance^9^, ^10^. Positive expression of microtubule‐associated protein 1 light chain 3I, II (marker of autophagy) in chemoresistant OS cells is higher than normal bone tissue. Chemotherapy induces autophagy in OS cells and the inhibition of autophagy has been shown to enhance chemosensitivity in animal models^11^. Metadherin (MTDH), popularly called astrocyte elevated gene‐1 (AEG‐1), is significantly involved in cancer development^12^. It shows increased expression in breast tumors^13^, pancreatic carcinogenesis^14^, and gliomas^15^. MTDH is known to induce multidrug resistance gene 1 (MDR1) expression and participates actively in autophagy and chemoresistance^16^. Pei *et al.* found that MTDH‐stimulated 5‐FU chemoresistance may be triggered through AMPK/ATG5 pathway‐activated autophagy^17^. Nonetheless, the MTDH‐mediated autophagy induction mechanism remains largely unknown in OS.

MicroRNA (miRNA) can be defined as non‐coding single‐stranded (22–24 nucleotides) RNA that regulate expression of genes through 3′‐untranslated region (3′‐UTR) binding of their target mRNA^18^. The miRNA regulates almost all cellular processes, and most protein‐coding genes with a large number of non‐coding genes by transcription. In tumors, miRNA play a pivotal role in cell division, self‐renewal, invasion, and DNA damage^19^. They play a dual role in genetics and epigenetics (inhibit/promote cancer) in various tumors^20^. MicroRNA‐22 (miR‐22) is located on the 17p13 chromosome and happens to be evolutionarily conserved^21^. This highly conserved microRNA plays a significant role in oncogenesis, cell maturation, as well as cell proliferation, especially during stress^22^. Previous research has demonstrated that MiR‐22 functions as a tumor suppressor in different malignancies, such as prostatic^23^, breast^24^, and gastric cancers^25^. Furthermore, miR‐22 was appreciably decreased in OS patients while its overexpression could suppress MG‐63 cells from dividing and metastasizing^26^. However, the role of miR‐22 and the underlying mechanism in regulating OS and chemoresistance are hitherto unknown.

These studies indicate that miR‐22 and its target MTDH may play an important role in osteosarcoma cell proliferation, autophagy, and chemotherapy resistance. However, the relationship between miR‐22 and MTDH‐mediated autophagy in chemotherapy resistance of osteosarcoma cells remain unknown. Thus, the aim of the present study was to investigate the expression levels of miR‐22 in osteosarcoma cells following chemotherapy and to analyze the association with chemotherapy resistance *in vitro*. We further explore the role of miR‐22 regulation of autophagy by targeting MDTH in OS and chemotherapy sensitivity.

## Methods

### *Cell Culture*

We procured human osteosarcoma cell lines (MG‐63) from the Cell Bank of the Chinese Academy of Sciences (Shanghai, China). The cells were proliferated in DMEM medium (DMEM, Hyclone, USA) containing 10% FBS (HyClone, USA), 100 U/mL penicillin, and 100 μg/mL streptomycin, and incubated at a temperature of 37°C with 5% CO_2_ atmosphere.

### *Cell Transfection*

We used Lipofectamine 3000 (Invitrogen Life Technologies, Carlsbad, CA, USA) for all transfection assays according to the manufacturer's instruction. We transiently transfected MG‐63 cells using negative control (NC) or miR‐22 mimic. Overall, 50 μL Opti‐MEM (Gibco, 31985‐070) was used to dilute 50 nM NC or mimic, which was then added with the diluted 3 μL lipo 3000, and the mix was maintained for 20 min at room temperature. Post‐incubation, we added them to a 6‐well plate (100 μL liposome transfection mixture). After incubation for 6 h, the medium was replaced by DMEM (Hyclone, USA) with 10% FBS. After 48 h of incubation, we harvested the experimented cells for downstream analysis.

### *Cell Proliferation Assay*

MG‐63 cells (5000/per well) were plated in a 96‐well plate. Each plate included a control group, an NC group, a cisplatin group, and a cisplatin+miR‐22 group. Each group was seeded in three 96‐well plates. The cells were incubated in an incubator for 24 h. After adding cisplatin according to the grouping, they were placed in an incubator for 6, 12, and 24 h. We added 10 μL of MTT (5 mg/mL) in each well, and the cultivated the plates for 3 h in the cell culture incubator. We added approximately 150 μL of DMSO to each well after removing the medium. An Absorbance Microplate Reader (Thermo fisher, Multiskan 51119000, USA) platform was used to measure the absorbance at 570 nm. We repeated the assay thrice. We cultured cells in a 6‐well plate containing 2 mL of 37°C pre‐warmed medium for colony formation assay. After 2 weeks, 4% paraoxymethylene was used for fixing cells and we used 0.3% crystal violet to stain them. We enumerated the colonies manually. The experiment was repeated three times.

### *Monodansylcadaverine Staining*

The MDC (Solarbio, G0170, Beijing, China), is a marker for autolysosomes and is used as a tracer of autophagic vesicles. Cells were fixed and incubated with MDC for 30 min in 37°C and then stained with DAPI. We used anti‐fluorescence quenching slide (to avoid light) for fluorescence study using a confocal fluorescence microscope (Zeiss, LSM710, Germany). MDC fluorescence levels were also were qualitatively evaluated by flow cytometry. Cells were stained with MDC for 30 min at 37°C, then MDC fluorescence levels were detected by flow cytometry in FL1 channel.

### *Flow Cytometry Assay*

Detection of the autophagy‐related protein LC3 expression change was qualitatively evaluated by flow cytometry (BD Calibur, San Jose, USA). We fixed the cells with 4% paraformaldehyde for a duration of 10 min, followed by three phosphate‐buffered saline (PBS) washes. PBS‐1% BSA solution was used for preparing 1:500 dilution of LC3 antibody. Thereafter, we incubated the cells for 2 h with the primary antibody at room temperature. The Alexa Fluor‐488 conjugated secondary antibodies were dissolved in PBS‐1% BSA solution to achieve 1:400 dilution, added to the fixed cells in the dark at room temperature (for 1 h). We analyzed the treated cells using flow cytometry on a BD FACS Calibur platform. Cellular fluorescence changes were detected in FL1 channel.

### *Electron Microscopy*

Cells were fixed overnight at 4°C in 2.5% glutaraldehyde solution, and then incubated in 1% osmium tetroxide for 1 h at room temperature, before immobilization with 10% gelatin and fixing of the cells with glutaraldehyde for 1 h at 4°C. Post‐dehydration in an increasing gradient of ethanol concentration (30%, 50%, 70%, 90%, 95%, 100%, 100%), the samples were immersed in epoxy resin. The samples were sectioned with a Leica UC6 (Leica, EM UC6, Germany) and observed under a transmission electron microscope (JEM1011, Japan).

### *Luciferase Experiment*

We polymerase chain reaction (PCR)‐amplified 3′‐UTR of MTDH and cloned this into a pmirGLO Vector to construct the reporter assay vector. We transfected 60% confluent MG‐63 cells of 60% in 24‐well plates with reporter vector using Lipofectamine 3000. We co‐transfected 5 ng of pRL‐SV40 Renilla luciferase construct (for normalization), the constructed wild type or mutant pmirGLO vector (100 ng), and either 100 ng of NC or miR‐22 mimic per well, respectively. We isolated cellular extracts at 48‐h post‐transfection, and evaluated luciferase activity with the help of the Dual‐Luciferase Reporter Assay system (Promega, USA).

### *Real‐time Polymerase Chain Reaction Assay*

We used TRIzol Reagent (Invitrogen, USA) to isolate total RNA from cells as per the given instructions from the manufacturer. The PrimeScript RT Reagent Kit (TaKaRa, Dalian, China) was used for conversion of RNA into cDNA; the miRNA Extraction Kit (Ribobio, Guangzhou, China) helped to achieve the same objective with respect to miRNA. Quantification of transcripts was attained by performing RT‐PCR using SYBR Premix Ex Taq (TaKaRa, Dalian, China). We used the ABI StepOne Plus Real‐Time PCR System (Applied Biosystems; Thermo Fisher Scientific) to perform quantitative PCR (qPCR) using the SYBR‐Green PCR Kit (TaKaRa, Dalian). The qPCR protocol involves following steps: (i) initial denaturation for 5 min at 95°C; and (ii) 40 cycles involving denaturation at a temperature of 95°C for 10 s, followed by annealing and extension at 60°C for 34 s. We repeated the experiments thrice. MiR‐22 primers used Bulge‐loop miRNA qRT‐PCR Kit (Ribobio, Guangzhou, China). The qPCR primers were as follows: beclin1, CCCGTGGAATGGAATGAGATTA (Sense) and CCGTAAGGAACAAGTCGGTATC (Antisense); LC3, CATGAGCGAGTTGGTCAAGA (Sense) and CTTTCTCCTGCTCGTAGATGTC (Antisense); Atg5, CTTCTGCACTGTCCATCTAAGG (Sense) and ATCCAGAGTTGCTTGTGATCTT (Antisense); MTDH, CAGTGGGATGTTAGCCGTAATC (Sense) and ACTCTTCTGCTGGTGCATTC (Antisense); and glyceraldehyde 3‐phosphate dehydrogenase (GAPDH), CTTTGGTATCGTGGAAGGACTC (Sense) and AGTAGAGGCAGGGATGATGT (Antisense).

### *Western Blot Analysis*

We washed the cells with pre‐chilled PBS once and lysed the cells in 200 μL RIPA buffer for 30 min duration by keeping the vials on ice and centrifuged at 13500g at 4°C for 5 min. SDS‐PAGE (Bio‐Rad) was used to resolve cellular proteins followed by blot transfer to PVDF membrane (Millipore, US). Post‐blotting, 5% skimmed milk was used for incubation of the membrane at 4°C for 1 h. We used 5% BSA‐containing TBST solution to prepare 1:1000 dilution of primary antibody and incubated the membranes overnight in it at 4°C. The membranes were washed thrice with TBST at room temperature for 10 min each time. The secondary antibody was diluted with TBST at 1:5000; membranes were incubated in it at room temperature for 1 h, followed by TBST washes at room temperature three times for 10 min each time; the chemiluminescence reaction was carried out with the Tanon chemiluminescence sensor system (Tanon, Shanghai, China). GAPDH was used as the loading control.

### *Statistical Evaluation*

We performed every experiment thrice and results were represented in the form of mean ± standard deviation. We used SPSS 20.0 software (IBM, Armonk, NY, USA) for statistical calculations. Difference between two groups was compared by independent‐samples *t*‐test. Difference among three or more groups was compared by one‐way analysis of variance (ANOVA). We set the cut=off for statistical significance as a *P*‐value <0.05.

## Results

### *Inhibition Effects of miR‐22 in Anticancer Drug‐induced Cellular Division*

**Figure 1:**
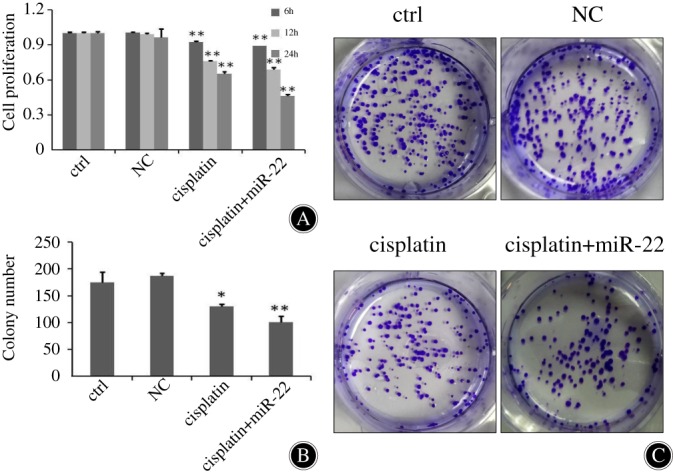
MG‐63 cellular proliferation was inhibited by MiR‐22. (A) The MTT assay performed for four groups of MG‐63 cells: viable cell count was evaluated through absorbance at 570 nm. Optical density (OD) was expressed in the form of mean ± standard deviation. The cisplatin + miR‐22 group significantly increased the inhibition rate induced by cisplatin (*P* < 0.01). (B, C) After transfection for 6, 12, and 24 h, the influence of miR‐22 on MG‐63 cell division was evaluated by colony formation assays, respectively. Cisplatin inhibits cell proliferation; adding miR‐22 enhances MG‐63 cell inhibition. *P < 0.05; **P < 0.01.

The MTT assay revealed that miR‐22 overexpressing MG‐63 cells had remarkable decline in proliferation capacity as opposed to the rest of the three experimental subsets (0.513 ± 0.001). MiR‐22 treatment significantly suppressed cell growth, while the negative control (NC) (1.070 ± 0.072) exhibited no effect on cellular proliferation when compared to untreated cells (1.109 ± 0.015). Transfection with miR‐22 gave rise to a marked decrease in osteosarcoma cell proliferation in the MG‐63 cell lines (Fig. 1A, *P* < 0.01). Similar to MTT results, the cell proliferation viability determined by colony formation assay of MG‐63 cells shows that cisplatin reduces cellular proliferation (129.6 ± 4.163) and it was further suppressed upon introduction of miR‐22 (101.0 ± 10.58). Cells treated with miR‐22 mimics formed a smaller number of colonies than the control group (174.6 ± 19.86, *P* = 0.004). Colony formation assays to test MG‐63 cells’ viability demonstrated that the miR‐22 group had a lower cell proliferation rate than the cisplatin group (Fig. 1B; C; *P* < 0.05).

### *Inhibition Effects of miR‐22 in Autophagy of Osteosarcoma Cancer Cells*

**Figure 2:**
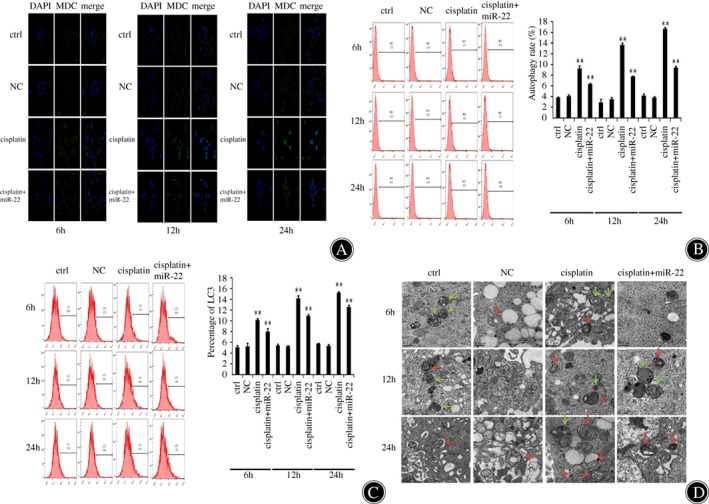
The miR‐22 inhibited autophagy in osteosarcoma cancer cells. (A) We detected the expression of monodansylcadaverine (MDC) in MG‐63 cells transfected with miR‐22 or negative control (NC) for 6, 12, and 24 h through fluorescence microscopy. MG‐63 cells were weakly positive in miR‐22 mimic‐transfected cells. Nuclei stained with DAPI appear in blue. (B) MDC staining was used to detect the extent of autophagy through flow cytometry. The results indicated that autophagy was markedly decreased in MG‐63 cells that were transfected with miR‐22. **P < 0.01. (C) The autophagy‐related protein LC3 expression was measured by flow cytometry at 6, 12, and 24 h after transfection. Right shift and fluorescence levels in the cisplatin + miR‐22 group increased, showing that autophagy inhibition was more significant in the cisplatin + miR‐22 group. **P < 0.01. (D) The transmission electron microscopy (TEM) images represent autophagosomal ultrastructures in cells transfected with miR‐22, followed by cisplatin (20 μM) treatment for 6, 12, and 24 h. Autophagosomes (red arrows) and lysosomes (green arrows) are represented in TEM images. TEM revealed a marked accumulation of autophagosomes in the cytoplasm of cisplatin group compared with the cisplatin + miR‐22 group.

We additionally wanted to confirm the importance of miR‐22 in the regulation of osteosarcoma autophagy. As shown in Fig. 2A, compared with the control group, there was an increasing number of MDC positive staining cells with attenuated staining intensity of the cisplatin group, and the groups of miR‐22 mimics inhibited a higher level of autophagy than the control group. Fluorescence microscope observation and flow cytometry assay using MDC staining show that the cisplatin + miR‐22 group (6.330 ± 0.132) had significant autophagy inhibition compared to the cisplatin group (9.217 ± 0.522). With prolongation of time, the right shift and fluorescence levels in the cisplatin + miR‐22 group (9.447 ± 0.225) increased, showing that autophagy inhibition was more significant in the cisplatin + miR‐22 group (Fig. 2B, *P* < 0.01). Consistently, onset of autophagy‐associated protein LC3 was visualized by flow cytometry. Cisplatin promotes the expression of autophagy‐associated protein LC3 (15.23 ± 0.153), and miR‐22 inhibits cisplatin‐induced elevation of LC3 expression in cells (12.57 ± 0.306) (Fig. 2C, *P* < 0.01). We used transmission electron microscopy (TEM) to assess autophagosomes. Untreated MG‐63 cells accumulated a large number of bulky autophagic vacuoles having quintessential double‐layered membranes with organelle remnants. In contrast, miR‐22‐transfected/cisplatin‐treated cells exhibited only a few vacuolar structures (Fig. 2D). The lysosomes were present in each group of cells. As time went on, autophagosomes slowly developed, and autophagy in the cisplatin + miR‐22 co‐treatment group was less than that in the cisplatin alone group. We observed an appreciable assimilation of autophagosomes in the cytosol of the cisplatin group cells in contrast to the cisplatin+miR‐22 group cells (Fig. 2D).

### *Functional Roles of miR‐22 in Sensitization of Osteosarcoma Cells to Cisplatin Treatment via Upregulating ATG5, Beclin1, LC3, and Metadherin*

**Figure 3:**
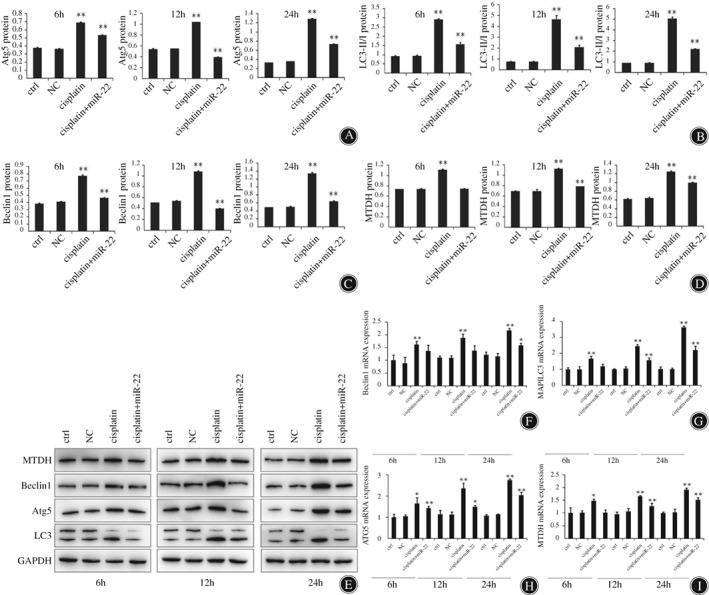
MiR‐22 enhances cisplatin sensitivity of osteosarcoma cells through autophagy regulation. (A–D) Western blotting helped to detect beclin1, LC3, metadherin (MTDH), and ATG5 expression in MG63 cells after 6, 12, and 24 h. Glyceraldehyde 3‐phosphate dehydrogenase (GAPDH) served as a loading control to check for uniform loading of protein in all lanes (E). *P < 0.05, **P < 0.01. (F–I) Expression levels of beclin1, LC3, MTDH and ATG5 mRNA were analyzed through quantitative reverse transcription polymerase chain reaction post‐miR‐22 treatment of MG‐63 cells. The results showed that miR‐22 mimics has reduced the level of beclin1, LC3, MTDH, and ATG5 in osteosarcoma cells. *P < 0.05, **P < 0.01.

From the above experimental results, we found that miR‐22 could suppress MG63 cell proliferation and autophagy. Thus, we would like to further explore the mechanism of these biological behaviors at the molecular level. The western blot analysis showed that autophagy‐related proteins beclin1, LC3, MTDH, and ATG5 had significantly lower expression in the miR‐22 + cisplatin group after 6, 12, and 24 h. The bioinformatics tool identified that beclin1, LC3, MTDH, and ATG5 were potential targets of miR‐22. We observed miR‐22 inhibiting beclin1, LC3, MTDH, as well as ATG5 mRNA expression. At 24 h, the beclin1, LC3, MTDH, and ATG5 of the miR‐22 + cisplatin group was 1.583 ± 0.097, 2.214 ± 0.233, 1.517 ± 0.081, and 2.035 ± 0.146, respectively; and the differences were significant (*P* < 0.01). Cisplatin promoted autophagy‐related gene expression, and miR‐22 overexpression inhibited autophagy gene expression, confirming that overexpression of miR‐22 contributes to anticancer drug‐induced autophagy in OS cells. (Fig. 3; *P* < 0.05; *P* < 0.01).

### *Functional Roles of miR‐22 in Sensitization of Osteosarcoma to Chemotherapeutic Agents’ in Vitro Metadherin Targeting*

**Figure 4:**
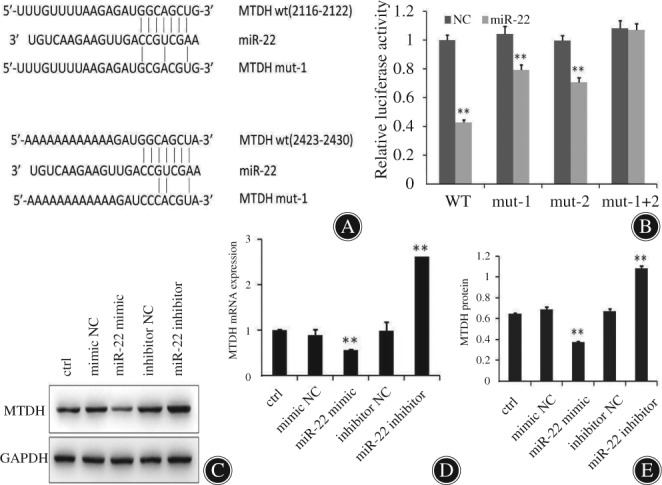
MiR‐22 regulates autophagy by targeting metadherin (MTDH) in osteosarcoma cells. (A) Luciferase assay on MG‐63 cancer cells, which were co‐transfected using mimics of miR‐22 and a luciferase reporter cloned with MTDH3′‐UTR (MTDH‐wt), MTDH3′‐UTR (MTDH‐mut1), and MTDH3′‐UTR (MTDH‐mut2), which harbored four nucleotide substitution within the binding sequence of miR‐22. We used empty construct of luciferase reporter to serve as the negative control. (B) The relative luciferase function in MG‐63 cells was assessed after co‐transfection of miR‐22 and MTDH 3′‐UTR or mutant plasmids. It can be seen from the figure that miR‐22 has two binding sites with MTDH 3′‐UTR. **P < 0.01. (C–E) miR‐22 regulation of MTDH mRNA and protein levels through polymerase chain reaction and western blot analyses. Transfection of miR‐22 mimic, miR‐22 inhibitor to decrease and increase the MTDH mRNA and protein. **P < 0.01; glyceraldehyde 3‐phosphate dehydrogenase (GAPDH) served as a positive control; WT, wild‐type.

Beclin1, LC3, MTDH, and ATG5 showed downregulation at both mRNA and protein levels in cells transfected with miR‐22 mimics. To further assess whether miR‐22 was directly targeting MTDH expression through the target site in the 3′‐UTR of MTDH, reporter constructs containing either the wild‐type (WT) MTDH 3′‐UTR or MTDH 3′‐UTR with mutation (MUT) at the predicted miR‐22 target sequence were co‐transfected into osteosarcoma MG‐63 cells and then transduction of control, mimic NC, miR‐22 mimics, inhibitor NC or miR‐22 inhibitor. However, in cells that were transfected with the vector harboring two mutations, luciferase activity remained unchanged. In contrast, luciferase activity was suppressed with mutations in one of the two sequences. The findings indicate miR‐22 targets MTDH‐3′‐UTR at two binding sites. We validated MTDH to be a direct target of miR‐22 through expression studies using qRT‐PCR and western blot experiments in osteosarcoma cells. Transfection of miR‐22 mimic significantly reduced MTDH mRNA (0.561 ± 0.013) and protein amounts (0.374 ± 0.005), whereas miR‐22 inhibitor treatment upregulated the expression levels (2.623 ± 0.002, 1.081 ± 0.023) compared to the control group (1.000 ± 0.024, 0.647 ± 0.005) (Fig. 4; *P* < 0.01).

## Discussion

Our study indicates that miR‐22 was successful in the inhibition of OS proliferation and autophagy, as well as increased chemosensitivity *via* MTDH inhibition. The results suggest that miR‐22/MTDH can act as a potential target for OS therapeutic treatment. The present research highlights that miR‐22 increases cisplatin sensitivity of OS cells *via* regulating apoptosis. Moreover, at the molecular level, beclin1, LC3, MTDH, and ATG5 mRNA and protein expression were inhibited by miR‐22 in MG‐63 cells. Beclin1, LC3, and ATG5 are a crucial autophagy‐related protein, which is involved in both autophagy and apoptosis^27^, ^28^, ^29^. MTDH has been observed to regulate proliferation and autophagy, as well as resistance to drugs^30^. Autophagosome development is found to be associated with the cytosolic‐associated protein light chain 3 (LC3), one of the mammalian homologues to yeast gene Atg5. It is involved in the early stage development of autophagosome through Atg12 with Atg8 conjugation^31^. Bioinformatic analysis identified that miR‐22 targeted MTDH. Luciferase reporter experiments proved that miR‐22 mediated repression of MTDH mRNA and protein expression.

We observed that miR‐22 in OS directly binds to MTDH mRNA 3′‐UTR. Our results proved that miR‐22 promotes the sensitivity of OS cells by downregulating autophagy regulated by MTDH. Thus, it is indicated that miR‐22 is a potential candidate as an adjuvant therapy to treat people suffering from OS. Our findings further expand on the significance of miR‐22 to the sensitivity of OS cells. MiR‐22 has already been found to act as either a tumor suppressor or an oncomiRNA in different cancers. Recent research found that miR‐22 promotes autophagy of human ovarian cancer cells through the suppression of the Notch signaling pathway^32^. In contrast, we observed miR‐22 to promote osteosarcoma chemoresistance *via* autophagy repression and stimulation of apoptosis, suggesting that miR‐22 functions in a disease‐specific context. In line with the tumor suppressive function of miR‐22 in OS autophagic chemotherapy resistance during the course of treatment which is mediated by HMGB1^33^, we demonstrated that miR‐22 can function as anti‐oncogene in the osteosarcoma development.

Multiple studies have revealed that microRNA regulate MTDH to induce chemoresistance^34^. Our results confirm that miR‐22 directly affects MTDH. MiR‐22‐enhanced production reduced the chemoresistance in OS cell lines. Increase of miR‐22‐mediated autophagy was due to the upregulation of MTDH. MTDH has been found to be engaged in diverse signaling pathways, like NF‐κB, PI3K/Akt, MAPK, and Wnt/β‐catenin. Overexpression of MTDH is observed in a variety of cancers belonging to all biological systems, and has crucial relevance in cancer progression, including proliferation, autophagy, and chemoresistance^35^. Several studies also highlight that MTDH increases autophagy using a non‐classical pathway. Expression of ATG5, AMPK phosphorylation, and consequent catalysis of autophagy are all induced by MTDH^36^, ^37^. Pei *et al.* noted that MTDH and autophagy‐related LC3‐II expression increased simultaneously. MTDH overexpression induced autophagy while its depletion suppressed autophagy^23^. Recently, MTDH has been recognized as an important mediator of tumors in chemoresistance and metastasis^13^, ^38^. *In vitro* and *in vivo* chemical resistance analysis has indicated that MTDH knockdown sensitizes several different cancer cell lines to paclitaxel, doxorubicin, cisplatin, and ultraviolet radiation^39^. The MTDH increases cell survival by promoting survival pathways such as PI3K and NF‐κB^40^, ^41^. Regulation of cell proliferation and metastasis *via* MTDH‐mediated autophagy is mediated by several miRNA, such as miR‐1297^14^, miR‐145^42^, miR‐98^43^, and miR‐217^44^. Our studies further revealed that miR‐22 increased the chemosensitivity through the miR‐22/MTDH/autophagy regulatory loop.

We found that miR‐22 had a significant effect on the chemoresistance of OS cells, as shown by the MTDH expression level. This may have affected the prognosis of OS patients. However, our results may be limited owing to increased variability reflected in miRNA expression from inter‐individual as well as race variation. Another limitation could be the use of limited OS cells from a cell line. Consolidation of the role of miR‐22 requires more research using an increasing number of cell lines and primary tumors. Delivery of miR‐22 inhibitor *in vivo* has to be optimized and its efficacy enhanced. Downregulation of miR‐22 and development of chemoresistance in OS requires more exhaustive work.

In a nutshell, the present research shows that miR‐22 is a critical regulator and that it suppresses autophagy by MTDH in OS cells during the course of chemotherapy, and induces sensitivity of OS cells to anticancer drugs *via* cell proliferation inhibition. We thus show miR‐22 to be valuable in the chemotherapy strategy for OS treatment.
